# Probability of COVID-19 infection by cough of a normal person and a super-spreader

**DOI:** 10.1063/5.0041596

**Published:** 2021-03-05

**Authors:** Amit Agrawal, Rajneesh Bhardwaj

**Affiliations:** Department of Mechanical Engineering, Indian Institute of Technology Bombay, Mumbai 400076, India

## Abstract

In this work, we estimate the probability of an infected person infecting another person in the vicinity by coughing in the context of COVID-19. The analysis relies on the experimental data of Simha and Rao [“Universal trends in human cough airflows at large distances,” Phys. Fluids **32**, 081905 (2020)] and similarity analysis of Agrawal and Bhardwaj [“Reducing chances of COVID-19 infection by a cough cloud in a closed space,” Phys. Fluids **32**, 101704 (2020)] to determine the variation of the concentration of infected aerosols with some distance from the source. The analysis reveals a large probability of infection within the volume of the cough cloud and a rapid exponential decay beyond it. The benefit of using a mask is clearly brought out through a reduction in the probability of infection. The increase in the probability of transmission by a super-spreader is also quantified for the first time. At a distance of 1 m, the probability of infection from a super-spreader is found to be 185% larger than a normal person. Our results support the current recommendation of maintaining a 2 m distance between two people. The analysis is enough to be applied to the transmission of other diseases by coughing, while the probability of transmission of COVID-19 due to other respiratory events can be obtained using our proposed approach.

Since the spread of coronavirus is through airborne transmission, studying events that can suspend the virus in air assume great significance.^1–7^ The exhaled breath from a potentially infected person can disperse the virus in the surrounding air through coughing, sneezing, laughing, moist talking, etc. The objective of our work is to assess the risk involved in some of these respiratory events with the present focus being on infection by coughing. We consider a typical scenario where a person in a closed space, such as a train/aircraft/restaurant/elevator/cinema hall, coughs with several people in the vicinity (Fig. 1). Similarly, when a caregiver approaches a patient and the patient suddenly coughs, it is important to understand the probability of transmission of the disease in order to better manage the ongoing unfortunate situation created by the COVID-19 pandemic. While the probability of transmission of disease in a closed space has been modeled in the past,^8–10^ the corresponding probabilities during various respiratory events have not been worked out and documented. The earlier studies assumed a homogeneous variation of concentration or quantum of infection inside the room. However, coughing leads to a much larger amount of contaminant within the cough cloud as compared to the surrounding air. This non-homogeneous variation needs to be properly modeled to understand the increase in the probability of infection with coughing.

**FIG. 1.**
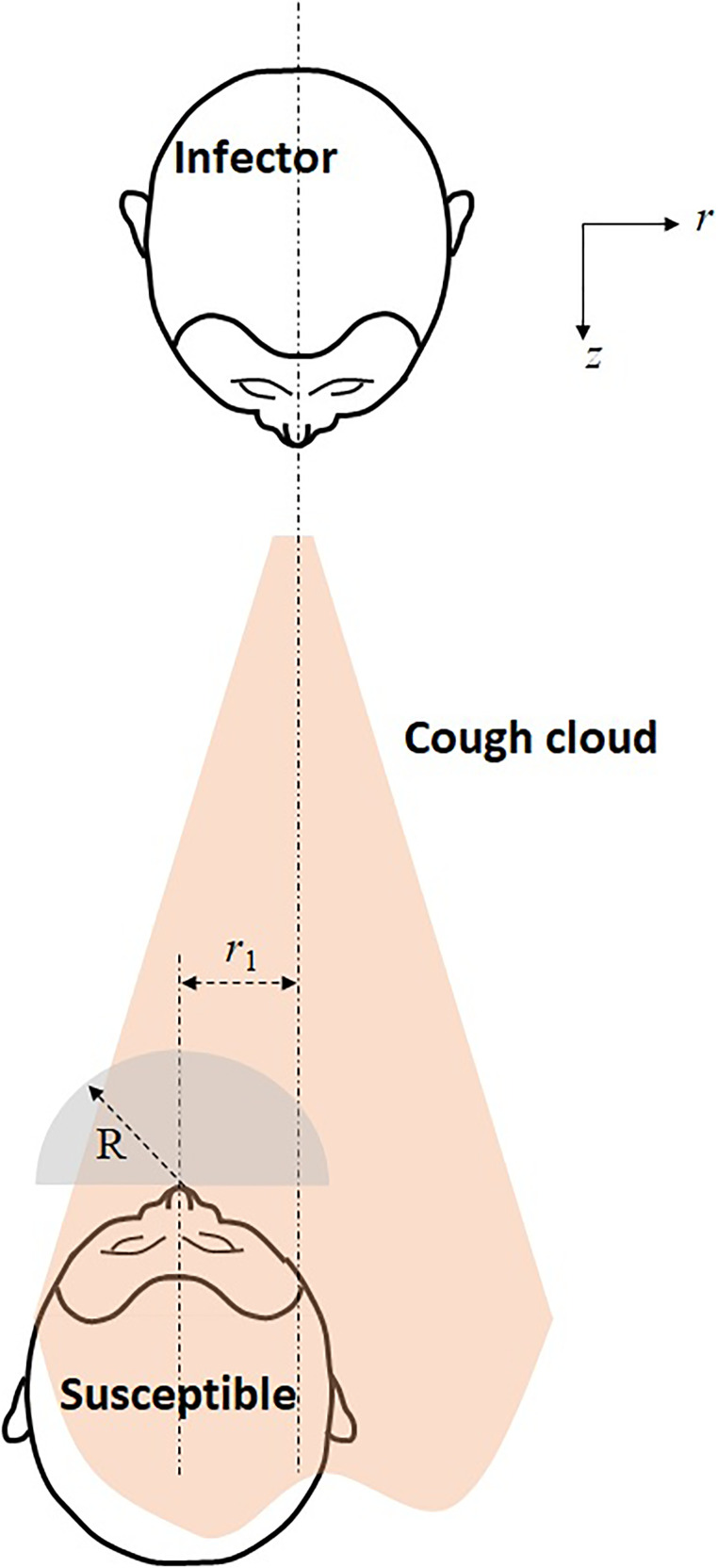
Schematic of the cough cloud generated by an infector and inhaled by a susceptible, positioned at an offset *r*_1_ from the center-line of the cloud. The volume of the infected air inhaled is approximated by a hemisphere of radius *R*.

In this context, the present work is among the first attempts to quantify the probability of infection during coughing. We also provide the spatial distribution of the probability close to the source in order to better understand the spread of the disease by coughing. We also evaluate the benefit offered by using a mask in terms of reduction in the probability of infection. We rely on the experimental data of Simha and Rao^11^ and the similarity approach of Agrawal and Bhardwaj^6^ to compute the spatial distribution of the concentration of infected aerosols in the cough-cloud, which is converted into a probability distribution of infection. The Wells–Riley equation is the cornerstone equation that has been employed in the past for computing the probability distribution, and the earlier approach is suitably adapted in the current work.

Several studies have examined the distance traveled by droplets ejected during various respiratory events and their drying time on various surfaces. These parameters help determine the transmissivity of the coronavirus and therefore assume great significance in the current times. For example, Chaudhuri *et al.*^12^ discussed the respiratory droplet physics and its potential connection with COVID-19. Das *et al.*^2^ examined the distance traveled by different sized droplets and recommend that the safe distance between people should be based on the distance traveled by large droplets ejected during various respiratory events. The effect of external conditions such as temperature, humidity, and wind flow was also examined. Pendor and Páscoa^13^ also found that the movement of the expelled droplets is influenced by their size, angle of ejection, velocity of the carrier fluid, and other environmental factors. Renzi and Clarke^14^ attributed the large range of droplet movement to the leading vortex ring. This relatively large and strong vortex helps to re-suspend the droplets as the droplets begin to settle out of the cloud due to their weight. Dbouk and Drikakis^1^ investigated the effect of wind speed on dispersion of droplets. They found that the droplets can travel beyond the recommended safe distance of 2 m with moderate wind speed. Cummins *et al.*^15^ found that the intermediate size droplets have the minimum horizontal range. Vadivukkarasan *et al.*^16^ identified the role of Kelvin–Helmholtz, Rayleigh–Taylor, and Plateau–Rayleigh instabilities in the breakup of the expelled respiratory liquid into respiratory droplets. Bhardwaj and Agrawal^17,18^ examined the drying of respiratory droplets under a variety of environmental and surface conditions. Bhardwaj and Agrawal^19^ pointed out that the long survival time of the coronavirus on surfaces, seen in some experiments, is due to the presence of a thin nanometric film that provides a hotbed for survival of the virus. Chatterjee *et al.*^20^ explained the shorter survival time of the coronavirus on porous surfaces as compared to impermeable surfaces seen in experiments.

The use of a face mask is one of the important recommendations to fight the pandemic. A number of studies have therefore examined the effectiveness of the face shield and face mask made of different materials. Sarkar *et al.*^21^ proposed a three-layer face mask with the inner and outer layer made of a hydrophobic material and the middle layer being hydrophilic. This design allows the high-momentum droplets to pass through the hydrophobic layer and get absorbed in the hydrophilic layer. Verma *et al.*^22^ found that homemade masks with multiple layers of fabric are effective in reducing droplet dispersal. The masks help by significantly reducing the speed and range of the respiratory jets. However, leakage through the mask and from small gaps along the edges should be avoided, as shown in a subsequent study by the group.^23^ This latter study also visualized the flow around a mask with an exhalation port. A large number of droplets were seen to pass unfiltered through the exhalation port, thereby reducing its effectiveness. Dbouk and Drikakis^24^ employed a computational fluid dynamics approach to study the effectiveness of masks during mild coughing events. Their study showed the benefit of using a mask, as it reduces the airborne droplet transmission both from the wearer to the surrounding and vice versa. Kumar and Lee^25^ undertook a review of various face masks and suggested designs from the viewpoint of flow resistance and thermal comfort. Akagi *et al.*^26^ examined the effectiveness of a face shield to sneezing. Their simulations suggested a finite chance of inhalation of the droplets carried by the sneeze through the gap between the face and the shield. Busco *et al.*^27^ proposed an approach for studying sneezing within the numerical framework, which can be extended to multiple sources of sneezing in a complex domain. Arumuru *et al.*^28^ performed experiments on sneezing while maintaining self-similarity with actual sneezing. They found that the sneeze can travel up to 25 ft, while the use of a three-layer mask substantially reduces the distance. Hossain *et al.*^29^ developed a method to measure the charge and filtration efficiency in N95 masks. They further showed that it is possible to recharge the masks and recover its filtration efficiency. Neelakandan *et al.*^30^ developed a system for disposing used face masks, as per the Indian Council of Medical Research (ICMR) guidelines.

The above survey shows that a good amount of information about the airborne transmission of the coronavirus and the effectiveness of using a face mask is now emerging. However, not many studies quantifying the chances of infection from respiratory events are available. Chaudhuri *et al.*^31^ developed a model for finding the probability of infection based on the droplet distribution being log-normal and showed the advantage of using a face mask. The present work further attempts to fill this void, with the focus being on coughing. We also comment on the role of super-spreaders in transmitting the disease. The work assumes significance, given the prevailing circumstances.

First, we present a mathematical model to determine the distribution of the concentration of infected aerosols in the cough cloud. As per Simha and Rao,^11^ the velocity of the cloud produced by coughing varies with the distance from the source as

$$\frac{U_{c}}{U_{o}}=\exp\left(-\frac{4.763z}{d_{c}}\right),$$ (1)

where *U*_*c*_ is the front velocity, *z* is the axial coordinate, and *U*_*o*_ and *d*_*c*_ are the reference velocity and length scales, respectively. The exit velocity of the jet at the mouth and the distance at which the velocity reduces to 1% of the exit velocity were taken as the reference scales. Equation (1) was obtained by Simha and Rao^11^ as a curve fit to the experimental data for velocity vs distance and applies to coughing even from different subjects. The cough cloud is a transient flow; the transient nature of the flow becomes apparent by converting the above equation into a position vs time relation as^6^

$$t=\frac{d}{U_{o}}\left[\exp\left(\frac{z}{d}\right)-1\right].$$ (2)

In a manner similar to the study of Agrawal and Bhardwaj,^6^ we assume that the flow exhibits self-similarity^5^ and the time-averaged velocity can be described as a Gaussian function as follows:^32^

$$\frac{U}{U_{c}}=\exp\left(-\frac{r^{2}}{b^{2}}\right),$$ (3)

where *U* is the axial velocity, *r* is the radial coordinate, and *b* is the velocity width (corresponds to the radial distance where the axial velocity drops to *e*^−1^ of the centerline velocity). The cough cloud grows linearly with distance; therefore, *b* = *cz*, where *c* is the dimensionless spread rate of the cloud. Based on data given in the study of Zhu *et al.*,^33^ we employ *c* = 0.1. Recently, Wang *et al.*^34^ have shown that the lateral variation of the streamwise velocity can be described as *U*/*U*_*c*_ = cosh^−2^(*r*/*b*_1/2_), where *b*_1/2_ is the radial distance where the axial velocity drops to half of the centerline velocity. This suggested hyperbolic cosine function and the Gaussian function employed here both fit the data equally well in the bulk of the jet,^32^ justifying the assumption of the velocity profile being Gaussian in nature. The transient nature of the flow gets reflected in the spatial variation of the velocity and concentration.

We further assume that the concentration of infected aerosols varies in a manner similar to the velocity distribution given by Eqs. (1) and (3), with the only difference that the concentration width is 1.2 times the velocity width.^35^ The flow of contaminant at any cross section of the cloud can be obtained by multiplying the concentration by the mass flow rate at that cross section as

$$\begin{array}{l} \bar{C^{.}}=\int_{0}^{\infty }2\pi UCrdr\\ =\int_{0}^{\infty }2\pi U_{c}\;\exp\left(-\frac{r^{2}}{b^{2}}\right)C_{c}\;\exp\left(-\frac{r^{2}}{b_{c}^{2}}\right)rdr. \end{array}$$ (4)

We use the cylindrical coordinate due to the axisymmetric nature of the cough cloud.

All the contaminants ejected by the infected person will, however, not be inhaled by the neighboring person. It is reasonable to assume that all the air inhaled by a person come equally from all sides; this spherical symmetry is only broken due to the presence of the person's body (Fig. 1). We, therefore, assume a hemispherical region (of radius *R*) in which a person is breathing. (For 0.5 L of inhaled air, the radius of the hemisphere is 0.062 m.) The amount of contaminant breathed in by the neighboring person is, therefore, given by

$$\begin{array}{l} C_{R}=ft_{b}\int_{r_{1}}^{r_{2}}2\pi U_{c}\;\exp\left(-\frac{r^{2}}{b^{2}}\right)C_{c}\;\exp\left(-\frac{r^{2}}{b_{c}^{2}}\right)rdr\\ =\frac{\pi b^{2}U_{c}C_{c}ft_{b}}{B}\left[\exp\left(-\frac{Br_{1}^{2}}{b^{2}}\right)-\exp\left(-\frac{Br_{2}^{2}}{b^{2}}\right)\right], \end{array}$$ (5)

where *r*_1_ is a measure of the offset with respect to the axis of the cough cloud where the neighboring person is located, *r*_2_ = *r*_1_ + *R*, *t*_*b*_ is the breathing time (taken as 2 s), *B* is a constant, and *f* is the area fraction intercepted. The last two parameters are calculated as follows:

$$B=\left(1+\frac{b^{2}}{b_{c}^{2}}\right),\;f=\frac{\pi R^{2}}{\pi \left(r_{2}^{2}-r_{1}^{2}\right)}.$$

Clearly, $C_{R}$ is a function of location (*r*_1_ and *z*).

For one quantum of infection exhaled by the infected person, only a fraction of this quantum is inhaled by the neighboring person. This suggests that we should normalize the actual amount of the contaminant inhaled with the contaminant at the source $C_{s}$,

$$C_{s}=QC_{0},$$ (6)

where *Q* is the volume of air coughed and *C*_0_ is the exit concentration. The quanta of infection inhaled is, therefore,

$$\mu =\frac{C_{R}}{C_{s}},$$ (7)

and the probability for infection is given as follows:^8^

p=1−exp(−μ). (8)

As explained by Wells (see the study of Rudnick and Milton^8^), for the quanta (*μ*) breathed in by a person, the chance of infection is given by a Poisson distribution. The probability of not getting infected is *e*^−*μ*^, while that of getting infected is (1 − *e*^−*μ*^), as given in Eq. (8) above. Equation (8), with an appropriate expression for *μ*, is also known as the Wells–Riley equation and has been frequently applied^8–10^ to find the probability of infection in a closed room.

The quanta of infection inhaled [Eq. (7)] is independent of the exit concentration *C*_0_ because both $C_{R}$ and $C_{s}$ depend on *C*_0_; however, $C_{s}$ retains dependence on the volume coughed *Q*. Since, the values at the source (*Q* and *C*_0_) are frequently not known, we suggest replacing $C_{s}$ in Eq. (7) by the maximum value of $C_{R}$ (which occurs at 0.3 m from the mouth for the conditions assumed in this work).

Second, we employ the model presented above to quantify the chances of the spread of COVID-19. We employ Eq. (8) to compute the probability of infection as a function of distance *z* and offset from the axis *r*_1_. The value of input parameters employed in the present calculations are documented in Table I. The values of *U*_0_ and *d*_*c*_ used in the calculations in the present study are taken from the data in Ref. 11 (Table II).

**TABLE I.** Value of relevant parameters employed in the present calculations.

Parameter	Value
Volume coughed, *Q*	1 L
Volume inhaled	0.5 L
Radius of inhalation, *R*	0.062 m
Duration of inhalation, *t*_*b*_	2 s
Number of people in the vicinity, *n*	6

**TABLE II.** Values of *U*_0_ and *d*_*c*_ used in the present calculations.^11^

	No mask	Surgical mask	N95 mask
*U*_0_ (m/s)	6	2	0.8
*d*_*c*_ (m)	1.5	1.2	0.25

Figure 2 shows the variation in probability with distance, with the two persons located on the axis of the cough cloud (i.e., the offset is zero). The probability reduces strongly with distance and is greater than 0.5 until the 0.5 m distance. The reduction in probability is because the contaminants are dispersed over a relatively large volume, owing to the conically expanding nature of the cough cloud, and an increasingly reduced fraction of the contaminant is inhaled by the neighboring person. There is a rather sharp reduction in the probability beyond 1 m corresponding to the maximum extent of the cloud.

**FIG. 2.**
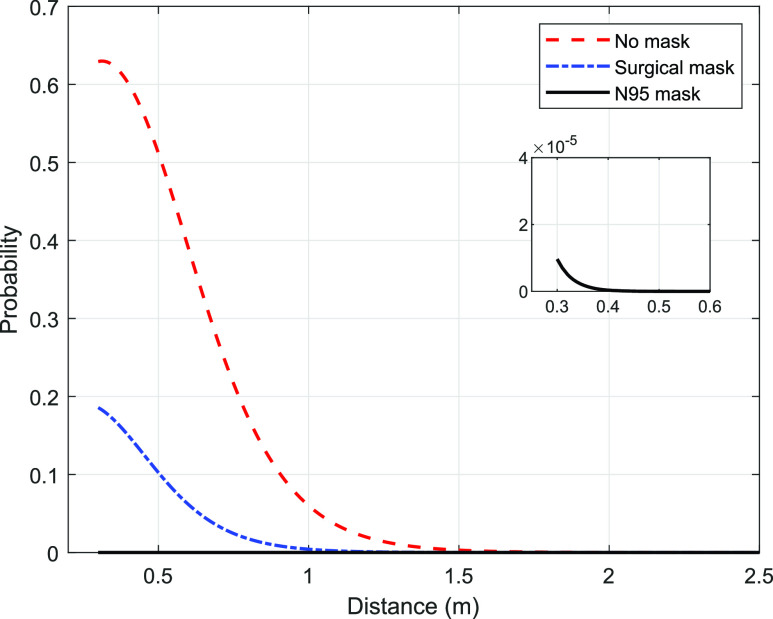
Distance-variation of the probability of the COVID-19 infection for three cases, namely, no mask, surgical mask, and N95 mask.

The same equation can be employed when the infected person or the neighboring person uses a mask, albeit with a different set of input parameters (Table II). For example, with a person using a mask, the chances of infection is reduced, and therefore, the source strength has to be appropriately reduced. At any given distance, the reduction in probability with the use of a surgical mask is clearly apparent from Fig. 2. With an N95 mask (shown in the inset of Fig. 2), the probability reduces to less than 4 × 10^−5^, which is less than that the expected value within a close space under normal condition. That is, with the use of an N95 face mask, the probability of infecting a person in the neighborhood reduces to the background value.

Figure 3(a) shows the variation in probability with distance and offset as a contour plot. A sharp reduction beyond the boundary of the cloud is again seen. In the lateral direction, the sharp reduction happens beyond about 0.06 m. For a person located close to the edge of the cough cloud, only a fraction of the inhaled air will be coming from the cloud, while the remaining fraction comes from the surrounding uncontaminated air. The fraction of the contaminated cloud air reduces drastically as the cloud boundary is crossed. The probability is relatively higher if the neighboring person is located within the cloud volume: more so, if the neighboring person is located within the 0.5 m distance. The plot gives the safe distance of at least 1.1 m that needs to be maintained, especially if one of the persons is a potential carrier and prone to coughing. This finding supports the commonly recommended distance of 2 m to be maintained between two persons.^36^

**FIG. 3.**
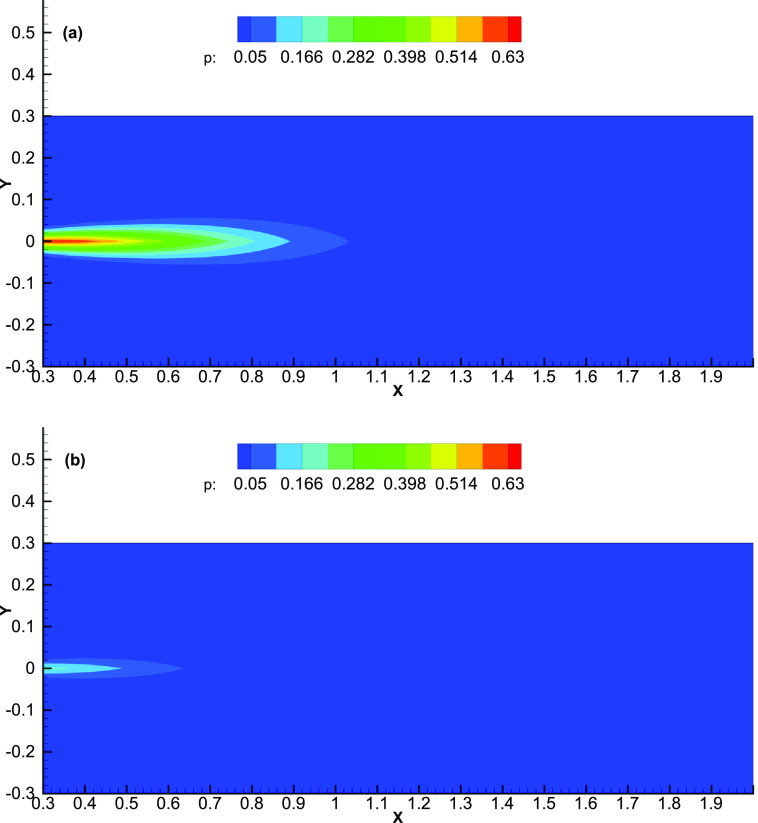
Color map of the spatial variation of the probability of the COVID-19 infection without a mask (a) and with a surgical mask (b). The same color-map is used in the figures to highlight the reduction in the chance of infection with use of a face mask.

Figure 3(b) shows that the probability reduces drastically with the use of a face mask. The chances of infection are reduced due to three factors: use of a mask by the person coughing reduces the source strength, the amount of contaminant dispersed is reduced, and the amount of contaminant inhaled by the neighboring person is reduced. The efficacy of using a face mask has further been confirmed through droplet flow visualization based experiments of Akhtar *et al.*^37^ for N-95, surgical, cloth PM 2.5, cloth, and wetted cloth PM 2.5 masks.

Third, we discuss the implications of the present results in understanding the increased or decreased risk of the transmission of COVID-19 and associated factors that influence the risk. The probability of infection is related to the quanta of contaminant inhaled by a person.^8,9^ For a closed space under the assumption of a homogeneous variation, the quantum of infection depends on^8^ the average fraction of indoor air that is exhaled breath, the number of infectors, the quantum generation rate, the total exposure time, and the number of people in the room. Here, we are able to describe the variation of concentration in a cough cloud and obtain an analytical expression for the probability of infection with coughing. We are able to work out a closed-form expression for variation in probability with location for a person coughing in a room, thereby proving that the probability of infection with a non-homogeneous distribution can also be handled within the theoretical framework. With a person coughing in the room, along the direction of the cough cloud, the probability increases until 63.2% from the baseline value. This increased probability applies for a few minutes; after this duration, the aerosols disperse over a much larger volume and the probability reduces and returns to its baseline value.

A few individuals may infect a large number of people, while several other infected people do not seem to pass on their infection to many others. In a recent study,^38^ the former category of “super-spreaders” (constituting only 5% of the infected population) were found to be responsible for 80% of the total infection, while 70% people did not pass on their infection to others. Besides extensive social contact, the super-spreaders tend to have an increased production of saliva, higher droplet load, and may shed the virus at a higher level.^39,40^ A super-spreader spreads the infection while shouting, talking in close proximity, and other such events.^40^

For a “super-spreader,” the strength at the source ($C_{s}$) is much larger than a “normal” person. This leads to a proportionately larger value of $C_{R}$ at a given location and a larger probability of infection. Figure 4 presents the probability variation for a super-spreader. A larger region is affected by the super-spreader as compared to a normal person, and the maximum probability in the cloud is larger by around 50% [compare Fig. 4 with Fig. 3(a)]. However, Fig. 3(a) and Fig. 4 are qualitatively similar, and similar comments as given above regarding the sharp drop beyond the cloud volume apply. For a super-spreader with three times the strength (C_0_ = 3), the probability of infection at *z* = 0.5 m increases by 72% from 0.514 to 0.885. The corresponding numbers at 1 m are 0.060 and 0.171 (almost a three-fold increase), respectively (Fig. 5). If the safe distance is considered to be the distance where *p* < 0.05, we get a distance of 1.03 m for a normal person and 1.22 m for a super-spreader. These numbers clearly highlight the additional risk that a super-spreader poses in spreading the infection.

**FIG. 4.**
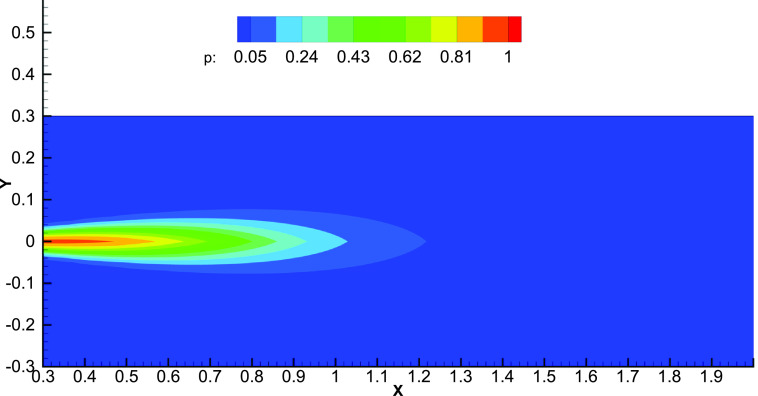
Color map of the spatial variation of the probability of the COVID-19 infection for a super-spreader. The source strength is taken as three times of the normal infected person.

**FIG. 5.**
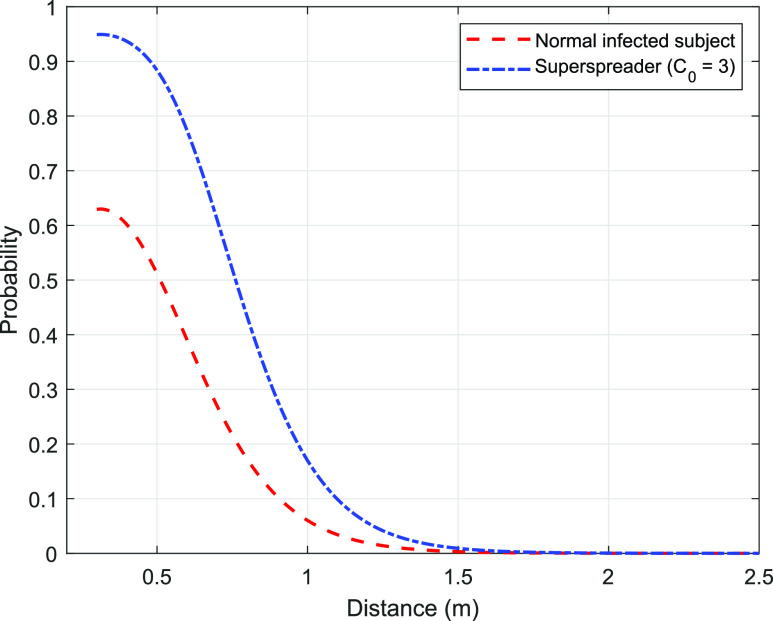
Comparison between the spatial variation of probability for a normal infected person and a super-spreader. The source strength is taken as three times of the normal infected person.

The model developed here for coughing can readily be extended to sneezing and other respiratory events. Since measurements with sneezing are far more difficult than with coughing, relatively gross parameters (such as the distance covered by the sneeze cloud) rather than the variation in local velocity and concentration are available.^27,28^ However, since the safe distance with sneezing needs to be estimated, we use the data of the probability of infection for coughing as follows: The probability of infection reduces to less than 5% for *z*/*L*_*c*_ = 0.8 (Fig. 2). Assuming that the probability of infection for coughing varies in a similar manner with the *normalized* distance for sneezing, we estimate the safe distance (i.e., probability < 5%) as 0.8 × 2.5 m^2^ = 2 m. Here, 2.5 m is the maximum distance traveled by the sneeze cloud.^5^

For other diseases transmitted by coughing, our model can be applied with the probability of infection appropriately adjusted. For example, if a larger quanta of contaminant is required for infection (say, equivalent to that ejected in three coughs), the quantum of infection in Eq. (7) is appropriately reduced (by a factor of three). Similarly, the model can be customized to a more (or less) susceptible person in the vicinity.

In summary, the present work focuses on understanding the transmission of the COVID-19 disease through coughing. We quantify the probability of infection for the first time for a super-spreader. Our modeling approach allows a simple and elegant way to estimate the variation in probability, in contrast to the computational fluid dynamics approach, where the results are case-specific and the overall approach is cumbersome.

The maximum probability of 63.2% at the source reduces exponentially to less than 1% over a distance of 1.5 m. A lateral distance of 0.1 m is recommended to be maintained from the person coughing in order to avoid infection. A surgical mask is found to reduce the chance of infection to about one-third of the no-mask case. The use of an N95 reduces the probability of infection to a negligible value. These results, therefore, quantify the benefit of using a mask during coughing.

The results are further extended to a super-spreader emitting thrice the quanta of infection compared to a normal person. The probability of infection is found to increase by 72% and 185% with respect to a normal person at distances of 0.5 m and 1 m, respectively, from the source.

Based on our analysis, we recommend simple measures, such as turning the head in a different direction, so as to avoid direct inhalation of the cough cloud to reduce the chances of infection. Similarly, the person coughing should turn their face away from other people to reduce the transmission of infection. Use of a mask, coughing in a handkerchief, coughing in an elbow are other measures that reduce the volume of the cough cloud and thereby the chances of infection. With sneezing, we estimate the safe distance to be 2 m.

The results presented here are significant as quantification of the probability of infection for various respiratory events is not previously available. Given the prevalence of coughing and its role in transmitting the coronavirus, the results are deemed to be significant. Our analysis can further be repeated and extended to other diseases and respiratory events.

## Data Availability

The data that support the findings of this study are available from the corresponding author upon reasonable request.
